# The quality of mental health care delivered to patients with schizophrenic disorder in the Italian mental health system. The QUADIM project A multi-regional Italian investigation based on healthcare utilization databases

**DOI:** 10.1192/j.eurpsy.2023.1039

**Published:** 2023-07-19

**Authors:** M. Monzio Compagnoni, G. Corrao, L. Allevi, A. Barbato, B. D’Avanzo, F. Carle, A. Saponaro, A. Gaddini, S. Scondotto, L. Ferrara, V. D. Tozzi, T. Di Fiandra, A. Lora

**Affiliations:** 1 Department of Statistics and Quantitative Methods; 2 National Centre for Healthcare Research and Pharmacoepidemiology, University of Milano-Bicocca, Milan; 3 Department of Mental Health and Addiction Services, ASST Lecco, Lecco; 4 Unit for Quality of Care and Rights Promotion in Mental Health, Istituto di Ricerche Farmacologiche Mario Negri IRCCS, Milan; 5 Center of Epidemiology and Biostatistics; 6 National Centre for Healthcare Research and Pharmacoepidemiology, Polytechnic University of Marche, Ancona; 7 General Directorate of Health and Social Policies, Emilia-Romagna Region, Bologna; 8 Agency for Public Health, Lazio, Region; 9 Department of Health Services and Epidemiological Observatory, Regional Health Authority, Sicily Region, Palermo; 10 Centre of Research on Health and Social Care Management, SDA Bocconi School of Management (Bocconi University), Milan; 11 Psychologist, previously General Directorate for Health Prevention, Italian Ministry of Health, Rome, Italy

## Abstract

**Introduction:**

The 1978 Italian reform of psychiatric services initiated the closure of psychiatric hospitals encouraging the development of community mental health. However, there is wide variability across regions in the amount of resources devoted to community-based psychiatric care, and the range of services provided still is cause of concern.

**Objectives:**

To evaluate the quality of mental health care delivered to patients with schizophrenia and related disorders taken-in-care by mental health services in four Italian regions (Lombardy, Emilia-Romagna, Lazio, Sicily).

**Methods:**

Thirty-one clinical indicators concerning accessibility, appropriateness, continuity, and safety were defined and estimated using healthcare utilization (HCU) databases, containing data on mental health treatments, hospital admissions, outpatient interventions, lab tests and drug prescriptions.

**Results:**

A total of 70,586 prevalent patients with schizophrenia treated in 2015 were identified, of whom 1,752 were newly taken-in-care. For most patients community care was accessible and moderately intensive. However, care pathways were not implemented based on a structured assessment and only half of the patients received psychosocial treatments. One patient out of ten had access to psychological interventions and psychoeducation. Activities specifically addressed to families involved a third of prevalent patients and less than half of new patients. One patient out of six was admitted to a community residential facility, and one out of ten to a general hospital psychiatric ward (GHPW); higher values were identified in new cases. In general hospitals, one-fifth of the admissions were followed by readmission within 30 days of discharge. For two- thirds of patients continuity of community care was met, and six times out of ten a discharge from a GHPW was followed by an outpatient contact within two weeks. For cases newly taken-in-care the continuity of community care was uncommon, while the readiness of outpatient contacts after discharge was slightly more frequent. Most of the patients received antipsychotic medication, but their adherence to long-term treatment was low. Antipsychotic polytherapy was frequent and the control of metabolic side effects was poor. The variability between regions was high and consistent.

**Conclusions:**

The Italian mental health system could be improved by increasing the accessibility to psychosocial interventions, improving the quality of care for newly taken-in-care patients, focusing on somatic health and mortality, and reducing regional variability. Clinical indicators demonstrate the strengths and weaknesses of the mental health system in these regions, and, as HCU databases, they could be useful tools in the routine assessment of mental healthcare quality at regional and national levels.

**Disclosure of Interest:**

None Declared

